# The Hospital Nursing Supplement

**Published:** 1893-07-08

**Authors:** 


"The Hospital" Nursing Mirror.

Being the Extra Nursing Supplement of "The Hospital" Newspaper.

[Contributions for this Supplement should be addressed to the Editor, The Hospital, 428, Strand, London, W.C., and should have the word "Nursing" plainly written in left-hand top corner of the envelope.]

### News from the Nursing World.

### THE VALUE OF A SIGNATURE.

The importance and significance of the letter which appears in another column signed Catherine J. Wood, can hardly be over-estimated. Any communication from the originator of a scheme would always receive consideration, and Miss Wood's able demonstration of what the charter now is, should command special attention. Her repudiation of a scheme by which a professional body proposes to "advertise its members" will be appreciated by all who wish to uphold the dignity of nurses. Miss Wood has the cordial concurrence of matrons as well as of employers of trained nurses in her assertion that "neither the Executive of the association nor its 'list' can guarantee the personal character and efficiency of a nurse; these qualities can be known only to those who have trained her and by whom she is personally known."

### PROVIDENT AND IMPROVIDENT.

Before the foundation of the Pension Fund, people used to say that nurses were improvident chiefly because they had no incentive to save. We may certainly endorse this opinion now, for a large number of nurses bear witness to its truth by the eagerness with which they flock to join the Royal National Pension Fund. Yet there will always be some improvident nurses of course, seeing that women are not all shaped in one mould mentally any more than physically. But the provident nurses are essentially liberal ones too. If any idea is put forward which seems to deserve support, such as temporary assistance for a disabled nurse (unhappily not a policy holder), the annual endowment of a nurses' bed, or a national holiday, we are certain to receive notes signed "Policy number —" and each one encloses some small subscription to the object under discussion at the moment. The Royal wedding does not call for any subscriptions from nurses, as those who know best have decided. The nurses will, of course, pay their own expenses for any organised fete, and these will be fixed at the lowest possible rate. But they are not desired to give to a wedding gift or to any object whatever save their own personal expenses. Neither provident nor improvident nurses ought to be taxed on an occasion which is solely one of national rejoicing.

### SICK-ROOM COOKERY.

The value of "kitchen physic" was understood by our grandmothers in spite of their ignorance of trained nursing, and doubtless their knowledge of cookery did much to help the weakly, despite other drawbacks, to regain health and strength. To-day sick folks can command skilled attendants, but they very often find that these do not possess the most elementary acquaintance with sick-room cookery. They may peptonise the patients' food, and understand the principle which governs this practice, but of the hundred and one ways of preparing dainty and appetizing nourishment they are grievously ignorant. The London Hospital probationers will have no opportunity of indulging in this modern ignorance of the kitchen department, the committee having arranged with the authorities of the National Training School for Cookery for courses of lectures to be delivered to the nursing staff on sick-room cookery. We congratulate Miss Lückes on a satisfactory arrangement, of which the nurses are gratefully availing themselves. The instruction comprises the preparation of all kinds of soups as well as beef tea, a great variety of jellies, the cooking of fish, chops, poultry, puddings of many kinds, invalid drinks of all descriptions, and other delicacies too numerous to enumerate. The course of twelve lessons is admirably planned, and embraces every detail which can serve to tempt the most capricious appetite.

### PRACTISING AND PREACHING.

Prevention is not only better but much easier than cure in cholera as in other diseases, and nurses should make it part of their daily duty to propagate this commonplace truth. Dr. Thorne Thorne's lecture and Sir Joseph Fayrer's, as well as the printed rules issued to the Queen's Jubilee Nurses and others on similar lines, the teaching of Mrs. Scharlieb, whose personal experience of cholera epidemics gives special value to her words, all these are valuable helps to nurses. If the latter will themselves teach, as well as practise, the strictest sanitary precautions they will be doing good work. District and private nurses have countless opportunities of giving useful hints to those whose knowledge of sanitary science is less than their own, and, if they do it with tact, their suggestions will always be well received. For themselves, indeed, "forewarned" is "forearmed," and we reckon on our English nurses showing in every possible way that they practise what they preach and preach what they practise, whether their duties include the care of cholera patients or are limited to more familiar epidemics.

### DISTRICT NURSING.

At a recent meeting of the Hampstead Nursing Association, an excellent record of work was laid before the subscribers. The ladies employed are all fully trained, and only attend on those persons who are too poor to pay for a private nurse. The district nurses act entirely under the orders of qualified practitioners, and the society is quite unsectarian. The management and supervision appear to be very good, and much local interest is taken in the movement. Several excellent speeches were made at the meeting, and the absence of Canon Ainger on account of indisposition was much regretted.

### PROVISIONS FOR CONVALESCENCE.

In connection with the Newcastle Cathedral Nurse and Loan Society, a Convalescent Home is shortly to be opened at Hexham. Good work having been already done by the little Home at Shotley Bridge, the society has every reason to hope for equal success in its larger venture. Much interest is taken by the workmen and others who benefit by the services of the trained nurses, seven of whom work under a lady superintendent in the districts around Newcastle. Night nurses are supplied when the urgency of cases demand them, and this is an advantage which certainly cannot possibly be over-estimated.

### A LOST OPPORTUNITY.

Men and women largely owe their success or failure in life to their own individual ability for availing themselves of opportunities. They seize or lose the chance which offers itself to them, perhaps only once in their career, and they rejoice over or lament "the fate" which they have unconsciously carved out for themselves. Amongst lost opportunities we must assuredly reckon one which is noted in the annual report of the Pembrokeshire and Haverfordwest Infirmary. Last summer Captain Rose generously offered his house and grounds, free of rent, rates, and taxes, as a Convalescent Home for the infirmary patients (especially the children) whom he also kindly promised to supply with milk, butter, fuel, and vegetables. The members of the board of management all agreed that such a noble offer must not be refused. But at their next meeting a month later they appear to have changed their minds, and Captain Rose's handsome offer was respectfully declined. Of course the universal complaint of want of funds was raised, there being a large balance due on the infirmary accounts of the previous year. It says little for the philanthropy of the neighbourhood that an independent convalescent fund was not started. Knowing what a desirable adjunct to a hospital or infirmary a Convalescent Home is proved to be, it seems short-sighted policy for any institution to lose such an opportunity as the liberality of Captain Rose placed before the good people of Haverfordwest.

### QUALIFIED MEDICAL WOMEN.

In 1884 the Queen personally commended the question of supplying medical aid to the women of India to the Countess of Dufferin, who was on the eve of departure for that country, The National Association for Supplying Medical Aid to the Women of India is the direct result of Her Majesty's action, and the society is, indeed, a worthy one. It is unsectarian, and it supplies fully qualified medical women—not young students whose friends consider that a year or two's training and a "vocation" enable English girls to treat native women in all emergencies. What disastrous results are obtained under the latter circumstance can only be adequately estimated by those whose personal experience has shown them how little supervised is the work of unqualified women in India. The work of the National Association for Supplying Female Medical Aid is thoroughly well organised, and the following duties of the Executive Committee call for general approval and support: (1) To bring the aims of the National Association prominently before the British public; (2) to aid in raising subscriptions for the Countess of Dufferin's Fund; (3) to select such lady doctors as may be required for the Association in India; and in this good work we wish them increased and continued success.

### SHORT ITEMS.

The Honourable Maud Stanley was prevented by illness from distributing the prizes to the students at the School of Medicine for Women on June 27th. Her place was taken by Mrs. Garrett-Anderson.—There are nearly three hundred officers, attendants, and nurses at the New London County Asylum at Claybury.—Ninety-five Russian women students are registered at the French medical faculties at present, only one Englishwoman, one German, one Turk, and twenty-five French.—Miss Bromley has recently given a very interesting course of lectures on "Health," which have been much appreciated by the members of the Y.W.C.A. at the Belgravian Institute.—Miss Hewett gave a lecture at the same institution last week, and succeeded in interesting her audience in her excellent practical hints for home nursing and for dealing with emergencies.—The new recreation ground of the London Hospital Clubs Union, situated at Lower Edmonton, was formally opened last week by Lady Clark. A large number of guests were present on the occasion.—The Guardians of the Coventry Union have raised the salary of Mrs. Dodd, the head nurse, from £30 to £35 per annum, and that of Nurse Whale from £25 to £30.—It is proposed to engage a second nurse to assist the one already doing useful work at St. Austell.—A very successful entertainment was held recently in aid of St. Luke's Home, Vancouver.

### THE DISABLED NURSE.

In a letter received last week from the "Disabled Nurse, she says: "I have at last been moved into lodgings near the sea. . . . I was carried here on a stretcher, and my back has been very painful since. I have been feeling worse in myself lately, but I have great hopes that the change will shortly do me good." The sum of £10 17s. 6d. has been forwarded to the "Disabled Nurse" in weekly instalments, leaving £3 3s. of the £14 0s. 6d. collected. As proposed in The Hospital of February 25th, we have forwarded £2 for the expenses of removal, and the balance of £1 3s. will be handed over in two payments for the weeks ending July 8th and 15th. We think that all our readers will be glad to remember how many payments they have enabled us to send in their names to the "Disabled Nurse."

### "THE HOSPITAL" ENDOWED BED.

We beg to acknowledge postal order for 1s. from the Rev. P. H. Good for the Hospital Endowed Bed.

### Sanitation for Nurses.

By a Sanitary Inspector.

### XII.—DISINFECTION AND DISINFECTANTS.

There is apt to be some confusion respecting the properties of disinfectants, antiseptics, and deodorants. The difference between a disinfectant and an antiseptic is rather one of degree than of kind. A true disinfectant is in reality a germicide, something capable of completely destroying both the germs of disease and the spores. It is invariably a strongly poisonous chemical agent, and it is essential for thorough disinfection that the solution shall be of sufficient strength and shall remain in contact with the germs for a sufficiently long time to thoroughly destroy both them and their spores, otherwise disinfectants are a delusion, and those who use them are merely sheltering themselves behind a false sense of security.

A real disinfectant does not necessarily destroy a substance in the sense that fire does, or some of the very strong acids, nor does it change necessarily the structure of the tissues subjected to its influence. An antiseptic, on the other hand, does not necessarily actually destroy germs, but checks their development, and makes them inert for the time being, at all events. A deodorant is a substance which overpowers other smells by substituting a stronger or different smell for the original one.

All sanitarians insist strongly on three conditions being observed if disinfection is to be a reality and not a farce. The first of these is the amount or strength of the particular disinfectant used. This must bear a proper proportion to the amount of the material to be disinfected.

Secondly, the disinfectant must be left in contact with the material long enough for it to be able to act as a germicide. It has been proved conclusively that even a weak solution of a disinfectant, if left in contact for a sufficient time, will thoroughly destroy germs.

Thirdly, the matter of temperature is important. Most germs can be destroyed at a sufficiently high temperature.

As regards chemical disinfectants, the most commonly used in a fluid form and those which have proved after repeated experiments to be the most effectual are corrosive sublimate or perchloride of mercury, having the chemical formula Hy. cl²., and carbolic acid. Other liquid disinfectants have been tried from time to time, such as chloride of zinc, commonly known as "Burnett's Fluid," and many others, but none have proved so satisfactory as the perchloride of mercury and carbolic acid.

Carbolic acid or phenol, which is prepared from coal-tar, is only useful as a disinfectant when the strength of the solution is 5 per cent., or 1 part in 20. "The solution generally useful is that obtained by making up one gallon of crude 80 per cent. acid to sixteen gallons with water." Dr. Wynter Blyth has found as the result of some careful experiments that whereas a 1 per cent. solution will destroy very feeble infectious germs, a 5 per cent. solution is needed to disinfect the more resistant forms of germ life, and the action must be prolonged before we can be perfectly certain that such germs are annihilated. The same authority says further: "If specific excreta are treated, it is doubtful whether 5 per cent. solutions are of sufficient strength, because associated with the hurtful material there is a quantity of organic matter which must on the one hand remove some of the phenol (or carbolic acid) from the sphere of action, and on the other impede the contact of the phenol with the substance which we wish to disinfect."

From this it is evident that the too common method in cases of infectious disease of dipping soiled linen requiring disinfection into a 5 per cent. solution of carbolic acid, and at once removing it, is of little or no use. The linen must soak in the solution for some time if the disease germs are to be effectually destroyed. Linen is merely dipped into the solution and then sent to the laundry, where it undergoes processes calculated to revive the germs, for unless the linen is absolutely boiled, warmth and moisture are just the conditions under which germs tend to flourish. Thus, unwittingly, all employed in the laundry to which such linen is sent are liable to a special danger of infection.

The same principle applies likewise to the disinfection of excreta. The method usually adopted is to pour the carbolic solution into the vessel containing the excreta, and then to proceed at once to empty away as rapidly as possible the whole contents of the vessel, and not giving the carbolic acid time to act on the infectious germs; the carbolic merely acts as a deodorant, and speedily becomes greatly diluted with the water in the drain, and the excreta enters the sewer absolutely as infectious and dangerous as before, and the carbolic acid has been simply wasted, and might just as well have never been added—or even better not at all, as then there would be no pretence of disinfecting. Likewise that favourite proceeding of nurses to pour a gallon or two of a disinfectant down a drain is as useless as it is wasteful. It is further stated with regard to carbolic acid that "owing partly to its volatility, which removes it in time, and partly to the peculiarity of its action, another danger attends its use in anything short of full strength and full doses when applied to kill contagion." The acid may, for a time, deprive the contagion of its infective power without permanently abolishing it, and the virulent properties may be regained whenever the acid has evaporated.

Corrosive sublimate is decidedly a more certain and effectual disinfectant than carbolic acid, in fact, many authorities consider it the most powerful disinfectant known. It has the great disadvantage of being extremely poisonous, but it has the advantage of being less bulky than carbolic acid. A solution of corrosive sublimate, 1 part in 1,000, is almost more effectual than the 1 in 20 solution of carbolic acid. Moreover, corrosive sublimate can be conveniently used as a 1 in 500 solution, whilst the strongest aqueous solution of this disinfectant which can be made is a 10 per cent. one. Some hydrochloric acid (or other free acid) should always be added to the solution of corrosive sublimate so as to prevent the formation of an insoluble substance called "albuminate of mercury," which is very apt to form a coat over the organic matter, and thus prevent the disinfectant from penetrating properly. Corrosive sublimate, like carbolic acid, must be left for some time in contact with the matter to be disinfected if it is to be really efficacious as a germicide. Moreover, both it and carbolic acid must be used in a concentrated form to thoroughly disinfect excreta.

A great advantage, from a nurse's point of view, that corrosive sublimate has over carbolic acid is that it does not cause the painful tingling and the numb feeling in the hands which carbolic acid of the 1 in 20 strength does, especially if the hands have to be immersed frequently in it, as they probably would have to be in order to disinfect thoroughly after infectious disease. Another drawback to carbolic acid, which is not shared by corrosive sublimate, is that its continued use causes the skin of the hands to crack, which is at once both painful and a source of danger to the nurse, as she thereby runs serious risk of herself becoming infected by the disease.

To make corrosive sublimate solution, it is recommended to dissolve half an ounce of corrosive sublimate in one ounce of hydrochloric acid, and add three gallons of water. As this disinfectant is extremely poisonous, and forms a colourless solution, without any odour whatever, it is necessary to give it a characteristic colour and odour, and this is best done by adding to the above solution five grains of aniline blue and one ounce of wood naphtha. In addition to this, the solution must be carefully guarded in every possible way, as giving an artificial odour and colour does not do away with its poisonous properties.

### The Royal Charter.

### ROYAL BRITISH NURSES' ASSOCIATION.

The following communications respecting the above Association will be of interest to our readers:—

The statements which have been made respecting the effect of the Charter granted to the Royal British Nurses' Association render it necessary that the members of the various hospitals and nurse training schools should clearly understand their position under the Charter. We, therefore, as representing the chief hospitals and nurse training schools of the metropolis which have taken part in opposing the Charter, think it right to call public attention to the important limitations which have been placed upon the powers originally sought for.

No opposition has at any time been raised to the incorporation of the Association, for the purpose of promoting such benevolent schemes for the benefit of nurses, as are now set forth in the Charter, as the first of the objects of the Association.

The opposition was directed mainly against the attempt to create a "list or register" which would be regarded by the public as a legally authorised "Register of Trained Nurses."

The Charter, as granted, substitutes for the "list or register of nurses," a "list of persons who may have applied to the Corporation to have their names entered therein as nurses, and whom the Corporation may think fit to enter therein from time to time, coupled with such information about each person so entered as to the Corporation, may, from time to time, seem desirable."

It is important, in order to prevent misunderstanding and to avoid misconstruction, that the following points should be clearly borne in mind:—

1. No professional privilege will be obtained by the nurses whose names appear upon the list.
2. The list will have nothing in common with legal registers of the medical or other professions, but will simply be a list of nurses published by the Association.
3. No nurse whose name appears on the list will have any right to use the title of "Registered Nurse."

It is desirable to add that a comparison of the draft Charter as submitted to the Privy Council, with the Charter as granted, clearly shows that the Privy Council have recognised the evils which might directly, or indirectly, have been occasioned by the establishment of a Chartered Register as originally proposed.

We are, Sir, your obedient servants,

Florence Nightingale.
Westminster, K.G., Chairman Metropolitan and National Nursing Association.
James G. Wainwright, Treasurer of St. Thomas's Hospital.
Henry Bonham Carter, Secretary Nightingale Fund.
T. S. Bristowe, M.D., F.R.S., Consulting Physician to St. Thomas's Hospital.
Seymour J. Sharkey, M.D., F.R.C.P., Physician and Lecturer to Nurses, St. Thomas's Hospital.
L. M. Gordon, Matron of St. Thomas's Hospital and Superintendent of Nightingale Training School.
E. H. Lushington, Treasurer of Guy's Hospital.
Thomas Bryant, F.R.C.S., Consulting Surgeon of Guy's Hospital.
E. C. Perry, M.D., Medical Superintendent Guy's Hospital.
Florence C. Nott Bower, Matron Guy's Hospital.
Rutherford Alcock, K.C.B., Vice-Chairman Westminster Nursing Home.
J. Troutbeck, D.D., Treasurer Westminster Nursing Home.
Mary E. Thynne, Hon. Sec. Westminster Nursing Home.
W. H. Alchin, M.D., F.R.C.P., Physician to Westminster Nursing Home and Hospital.
Thomas Bond, F.R.C.S., Surgeon to Westminster Nursing Home and Hospital.
Mary J. Pyne, Matron Westminster Hospital and Nursing Home.
John J. Hale, Chairman London Hospital.
Fredk. Treeves, F.R.C.S., Surgeon London Hospital.
A. Ernest Sansom, M.D., F.R.C.P., Physician London Hospital.
Eva C. E. Luckes, Matron London Hospital.
Henry Wace, D.D., Chairman of Committee of Management King's College Hospital.
John Curnow, M.D., F.R.C.P., Lecturer to Nursing Staff and Dean of Facility of Medicine to King's College Hospital.
C. Austin Leigh, Vice-Chairman King's Coll. Hospital.
Katherine H. Monk, Matron, King's Coll. Hospital.
John B. Martin, Treasurer, Charing Cross Hospital.
Fredk. Willcocks, M.D., Physician and Lecturer to Nurses Charing Cross Hospital.
H. A. C. Gordon, Matron Charing Cross Hospital.
W. A. Mackinon, K.C.B., Director Gen. Army Med. Dep.
W. Rathbone, M.P.
John R. Lunn, Med. Sup. and Lecturer to Nurses Marylebone Infirmary.
Elizabeth Vincent, Matron Marylebone Infirmary.
Alex. Marsden, M.D., Royal Free Hospital.

### MISS CATHERINE J. WOOD'S OPINION.

I have only to-day seen the full text of the Royal Charter of Incorporation granted to the Royal British Nurses' Association; though I have no longer any official connection with that body, I venture, as one of the signatories to the petition for incorporation, to ask leave to make a personal statement. There is a clause in the charter which was not in the draft read at the meeting at which I was present, nor was it embodied in the petition which I signed. It is as follows: (Purposes and Powers of the Corporation, Clause 2).—'The maintenance of an office or offices for supplying information to persons seeking for nurses, and to persons seeking for employment as nurses." This clause would never have received my assent. It places the Association, which was originated as a professional union, in the position of a Registry office or a Nursing agency; if the Association intends to be a medium for supplying the public with nurses (and it is difficult to put any other construction on these words) it is undertaking a work for which it cannot be qualified. Neither the Executive of the Association nor its "List" can guarantee the personal character and efficiency of a nurse; these qualities can be known only to those who have trained her, and by whom she is personally known. The "List" as a guaranteed record of a nurse's training and subsequent experience will eventually recommend itself to the public, and all the sooner if its authors abate their claim to omniscience; but it is surely beneath the dignity of a professional body to become a medium for advertising its members.—Trusting to your courtesy to insert this explanation. I am, Sir, yours faithfully,

Catherine J. Wood.

27, Percy Street, W. June 24th, 1893.

### Appointments.

[It is requested that successful candidates will send a copy of their applications and testimonials, with date of election; to The Editor, The Lodge, Porchester Square, W.]

Chorley Cottage Hospital.—Miss Mary Noble has been elected Matron of the Chorley Cottage Hospital. She was trained at the Bristol General Infirmary, and afterwards worked at the Children's Hospital, Bradford, and at the General Infirmary, Northampton. We congratulate her on her appointment.

Moseley Hall Convalescent Home for Children, Birmingham.—Miss A. Baugham has been appointed matron of this home. She was trained at the Borough Hospital, Birkenhead, and has since taken temporary charge of several hospitals and other institutions. We wish Miss Baugham success in her present post. Her testimonials are excellent.

### Where to Go.

The Obstetrical Society's next examination for midwives will take place on Wednesday, July 12th.

Mrs. Scharlieb, M.D., gave a lecture last Friday at the Trained Nurses' Club, which was well attended. Mrs. Scharlieb impressed upon her audience the need for thorough antiseptic precautions in all cases, and gave much valuable instruction on the subject of "Hœmorrhage, its Causation and Treatment."

### The International Nursing Congress.

(BY OUR SPECIAL CORRESPONDENT.)

THE International Nursing Congress at Chicago, of which Miss Isabel A. Hampton is chairwoman, has proved to be a brilliant success. Not only were the meetings of the Congress largely attended—it being frequently the case that some hundreds were present—and that amongst them were the representative lady superintendents of the great training schools for nurses and hospitals of the United States and Canada, in addition to representatives of many European nations, but the quiet, orderly, and business-like proceedings of the meetings impressed everyone favourably.

Miss Hampton is to be congratulated upon this excellent result of the first attempt to assemble a Congress of representatives of nursing throughout the civilised world. To her intelligence, capacity, and industry must be credited the larger portion of the undoubted success secured. By a fortunate coincidence Miss Hampton's new book, "Nursing: Its Possibilities and Practice," came to hand on the second day of the Congress. This book, which will be procurable in England shortly, represents a new departure in nursing literature. Heretofore, writers who have endeavoured to produce text-books for nurses have shown a disposition to go outside of nursing questions altogether, and to supply copy for their works by introducing many text-book extracts on such subjects as physiology, anatomy, materia medica, obstetrics, and chemistry. Miss Hampton's book extends to nearly 500 pages, but it faithfully adheres to its title, and is nursing from the first to the last page. We shall, of course, take an early opportunity of reviewing this excellent work; meanwhile, we ask for it a hearty welcome at the hands of every intelligent man and woman who is directly or indirectly connected with nursing in its best and widest sense.

### SOME CHARACTERISTICS OF THE CONGRESS.

Much instruction and some amusement were derived from the meetings of the Congress by those who had the wit to see and the experience to benefit. The discussions that followed the papers were throughout well sustained. They were remarkable from the precision and directness of the utterances of the majority, at any rate, of the speakers. The most curious, and on the whole probably the most interesting group represented—though a small one—was that of the advanced advocates of woman's rights, which considered any woman who accepted assistance, or counsel, or aid of any kind from anyone, ought to be unreservedly condemned as a hindrance and impediment to the progress of her sex. The words, "patronage," "charity," or "pecuniary aid" in any form to the ladies of this section, were anathema. They hold that, like the martyrs of old, the representatives of women workers in the nursing field, must be prepared to shed their blood, and if necessary to undergo hardships of the most severe and painful kind, so that they may emphasize and drive home the principle, that once a woman worker, and especially a woman worker in the nursing field, everyone must understand that they cut themselves adrift from aid of every kind, from every friendship of every kind, and every advice and every counsel of every kind, except such as they can derive from their individual perception, efforts, and mother-wit.

These children of fathers who have encouraged women to exert themselves in their own interests, are undoubtedly well-meaning, and we have neither the purpose nor the inclination to quarrel with them. Like the newly-qualified practitioner or the lawyer who has just passed the final examination, they are impressed with the feeling that they know everything and that the world lies open at their feet. Ten years' practical experience will do more to harden and strengthen, to modify and build up, these earnest representatives of the doctrine of "thorough," than anything else. No doubt their experience will teach them by the time they arrive at forty years of age that there is nothing so satisfactory to the human, whether man or woman, as the enlightenment which experience brings, in the sense that it reveals to every man and woman of intelligence that when they have learned everything it is possible to learn in this world, they become at last convinced of the profoundness of their own ignorance. There is no degradation in such a reflection, but a stimulating, earnest uprising of the spirit of the individual who humbly but earnestly pursues the paths which Providence has laid down for each to tread with an increasing usefulness and a sustained purpose which history shows has done more than anything else to raise and elevate the whole tone of society in every country and age. The earnest and advanced spirits we refer to will ultimately come to see that there is no greater privilege in this world than that of rendering to others such personal service as may be possible. Whilst he or she watchfully husbands the treasures which these paths reveal we may patiently await the awakening of those who will ultimately realise the value, the safety, nay, the necessity and the honour of being identified in the world's battle with such whole-hearted friends and colleagues. We have said more than we intended on this feature of the conference, and must now pass on to consider others.

### A PRACTICAL SPIRIT.

It is satisfactory to find that there is a practical spirit at work throughout the whole of the nursing world in these days, and that the ladies who have devoted their lives to this calling, when assembled in Congress, display qualities of business which do them high honour. The majority of those present were practical women, imbued with a high sense of responsibility, conscientiously anxious to do everything that was necessary to place nursing upon a proper and secure basis. There were a great number of this class present whose utterances displayed judgment, courage, knowledge and power. They were, of course, the backbone and foundation of the meeting, and they won for themselves and for the cause of nursing a higher place than has ever been obtained heretofore, either in public opinion or in the counsels of the world. We accord them our heartfelt and earnest thanks, and are glad to know that amongst them were representatives from England, and especially Miss Hughes, whose paper and contributions to debate were recognized as of great weight and importance. Miss Hampton had a most difficult duty to discharge. It was especially difficult to organize and bring to a successful issue the first International Congress of Nursing, and the thanks of all who have the best interests of nurses and nursing at heart are due to her for the triumphant success which the Congress achieved under her able guidance.

### THE POSITION OF MRS. BEDFORD FENWICK.

Our visit to Chicago and the inquiries made and information gained there, convince us that Mrs. Bedford Fenwick has not been quite fairly treated. She won golden opinions for herself at Chicago by the dignified and womanly way in which she discharged the difficult duties which devolved upon her in connection with the Woman's section of the World's Fair. There is no doubt that she had a considerable task to perform, that circumstances made this task of special difficulty, and that her great ability carried her successfully through, to the great satisfaction of all English people who had an opportunity of observing her conduct. Further than this, it is fair to her to state that on her first visit to Chicago she suggested that an International Congress of Nursing should be held in connection with the World's Fair. It was further suggested that Mrs. Bedford Fenwick should be the Chairwoman of such a Congress, but when the scheme was organised it was found undesirable to carry out the Congress on the lines which were first thought of. It was due to Mrs. Bedford Fenwick that she should have received an intimation directly this change of plan was decided upon. From causes which there would be no advantage in indicating, Mrs. Bedford Fenwick received the first information of the change, we believe, from the notices which appeared in The Hospital. This, to say the least, was unfortunate, and we feel it to be just to Mrs. Bedford Fenwick to state these facts as some justification for the statements which were made, presumably on her authority, both in the paper which she read before the Royal British Nurses' Association and in letters which were published at the commencement of the present year. The world progresses by differences of opinion, but these differences must never be allowed to interfere with justice to those who differ from us, and we have, therefore, much pleasure in making this statement in defence of a lady whose ability is undoubted, and who rendered most excellent service to hospital administration during the period she held office as Matron of St. Bartholomew's Hospital.

Mrs. Bedford Fenwick was not present at the International Congress, nor was her paper or the papers of Princess Christian, Mrs. de la Pledge, and others forthcoming. In this connection we congratulate Miss Lumsden, the Lady Superintendent of the Aberdeen Royal Infirmary, upon having sent an excellent paper upon nursing in Scotland.

### Subjects Dealt With.

The papers from representatives of nursing schools from the United States, Great Britain, France, Germany, Switzerland, Italy, Japan, and China, were so numerous as to make it very difficult to get through the whole of the programme in the short space of one week. English nursing was represented in excellent papers. On "The Training Schools in Great Britain," by Miss A. C. Gibson, Matron of the Birmingham Poor Law Infirmary; on "The Royal National Pension Fund for Nurses," by Miss Gordon, Matron of St. Thomas's Hospital and Superintendent of the Nightingale Training School for Nurses; "On District Nursing in England," by Mrs. Dacre Craven; "On the Work of the Queen's Jubilee Institute," by Miss A. Hughes, Superintendent Metropolitan and National Nursing Association; and on "Midwives," by Mrs. H. Smith, President of the Midwives' Institute. A paper promised by Miss Louisa Twining, on "The Workhouse Nurses' Association," did not come to hand, which was much regretted by the members of the Congress.

The United States contributed the following papers, most of which were of special value: "Training Schools in America," by Miss Irene Sutliffe, Superintendent of the New York Hospital School for Nurses; "Proper Organisation of Training Schools in America," by Miss Louise Darche, Superintendent New York City School for Nurses; "Nurses as Heads of Hospitals," by Miss E. P. Davis, Superintendent University of Pennsylvania Hospital; "Needs for an American Nurses Association," by Miss Edith Draper, Superintendent of the Illinois Training School for Nurses, Chicago; "Alumnæ Associations for Nurses," by Miss Isabel Merritt, Superintendent Brooklyn City Hospital School for Nurses; "District Nursing in America," by Miss C. E. Sommerville; "Children's Hospitals," by Miss Rogers, of Washington; and on "Obstetrics," by Miss Pope, also of Washington; "The Nursing of the Insane," by Miss May, of Rochester; "The Association of the Training of Attendants," by Miss D. H. Kinney, of Boston; and "Training Schools for Small Hospitals," by Miss M. Greenwood, of Cincinnati.

The most weighty contribution was, of course, that of Miss Hampton, on "The Standards of Education for Nurses." This important paper, which we hope will be published separately and widely circulated, was read in the general Congress, and elicited warm and well-deserved applause. A good paper was also read by Miss L. L. Dock, on "The Relation of Training Schools to Hospitals," and another on "Nurses' Homes," by Miss Lett, of St. Luke's Hospital, Chicago. The latter paper elicited a great deal of discussion, and created much interest.

France contributed papers on "Paris Free and Paying Hospitals," by Dr. Allen Herbert and Dr. W. Douglass Hogg, of Paris; on "French Nursing," and "French Training Schools," by Dr. L. N. Worthington and Dr. Leon La Forte respectively, both of Paris.

Germany contributed papers on "Trained Nursing in Berlin," by Fraulein Louise Fuhmann, of Berlin; "The Education of Nurses in Catholic Religious Orders of Nurses of Germany," by Sani Talhroth and by Dr. Kollen, Physician-in-Chief of St. Hedwig's Hospital, Berlin; and "The Work of Deaconesses in Germany."

Holland was represented by a paper on "The Systems of Hospital Nursing in Amsterdam," by Edouard Stumpff.

Switzerland, by a paper by Dr. Chas. Krafft, Lausanne, Switzerland.

Italy by a paper on "The Nursing Work of the Religious Orders of the Roman Catholic Church," by Cardinal Gibbons.

Japan and China, by a paper on "Missionary Nursing," by Miss L. Richards, of Boston, Mass.

In addition to these subjects, there were papers on "Nursing in Infectious Diseases," by Miss Drown; upon "Hospital Plans"; on "Military Movable Hospitals in India," "Army and Navy Hospitals," "The Marine Hospital Service," "Hospitals for Infectious and Contagious Diseases," "Obstetric Hospitals," "Diet Kitchens and Hospital Dietaries," and many other subjects. The two last papers, by Dr. P. H. Stehman and Miss Bolan respectively, were full of interesting matter. The solid, practical usefulness of these two papers, and especially of that by Miss Bolan, cannot be questioned.

### General Features.

The most noticeable feature of the Conference was the earnestness and dispatch with which much important business was disposed of. Everyone was transparently interested in the subjects discussed, and we have never attended a Congress which displayed more earnestness and good temper than that which was successfully brought to a conclusion on Saturday, the 17th ult.

It is understood that Mrs. Bedford Fenwick proposes to set to work to organise a Congress of Nurses in London next year. We would suggest that she should at once place herself in communication with the heads of the principal training schools and hospitals throughout the country, who should be invited to express an opinion as to the desirability or otherwise of making this attempt. It would be harmful to nursing to hold a congress which would only be representative of a portion, and that the smaller part of those responsible for the nursing of the sick in the United Kingdom. There can be no doubt that sooner or later it will be an advantage to hold an International Congress of Nurses in London. In order to secure the success of such an undertaking, it is, however, essential that the time for holding it shall be well chosen, and that all who are entitled to speak with authority on nursing matters, should be represented upon the council charged with the duty of organising such an assembly of nurses in England.

### Presentations.

On resigning her post as sister at the West London Hospital, Miss Alice Newport was presented by the nurses with a handsome afternoon tea service.

Miss Marriott, who has held the post of Lady Superintendent at the Memorial Cottage Hospital, Paulton, Bristol, for the last ten years, has been presented with a purse containing over £36, subscribed by her numerous friends in the neighbourhood.

### Everybody's Opinion.

*[Correspondence on all subjects is invited, but we cannot in any way be responsible for the opinions expressed by our correspondents. No communications can be entertained if the name and address of the correspondent is not given, or unless one side of the paper only be written on.]*

### A HOLIDAY HOME WANTED.

"R" WRITES: I advise "B. M. B." to write to the Matron, Convalescent Home, Cromer, for particulars, stating if she is willing to pay a small fee for use of Nurses' Rest Room.

### SMALL-POX.

"S. E. R." WRITES: In THE HOSPITAL last week I read, in a paragraph headed "Plain and Unattractive Duties," that no nurse could be found for a case of small-pox at Pershore. I think one would soon have been forthcoming if the want had been made public. I am a hospital trained nurse holding good certificates, and am at present at liberty.

*** Advertisements appear every week showing the urgent need for good nurses to work in infectious hospitals. Our correspondent should apply for a vacant post in one of these. —ED. *T.H.*

### "PLAIN AND UNATTRACTIVE DUTIES."

THE HON. SEC. OF THE WORCESTER INSTITUTION FOR TRAINED NURSES writes: My attention has been called to a paragraph in THE HOSPITAL Nursing Supplement for the 1st inst. headed "Plain and Unattractive Duties." The paragraph clearly implies that the case at Pershore therein referred to was not nursed by this Institution, because our nurses, in common with those of other kindred institutions, avoid small-pox and scarlet fever cases. As regards nursing institutions generally, I believe this insinuation to be absolutely groundless, and as regards the Institution at Worcester, it is capable of easy refutation, in proof of which I subjoin a list of infectious cases which have been nursed by us in the course of the present year. We did not undertake the Pershore case simply because we had no nurse disengaged at the time.

1893.

| | | | | |
|---|---|---|---|---|
| Jan. | 9 ... | Small-pox ... | 5 weeks ... | Nurse A |
| Feb. | 2 ... | Scarlet Fever ... | 4 " ... | " B |
| " | 23 ... | " " ... | 3 " ... | " C |
| Mar. | 3 ... | Small-pox ... | 3 " ... | " B |
| " | 3 ... | Scarlet Fever ... | 5 " ... | " D |
| " | 22 ... | Diphtheria ... | 4 " ... | " A |
| " | 28 ... | Small-pox ... | 6 " ... | " C |
| " | 28 ... | Scarlet Fever ... | 2 " ... | " E |
| " | 29 ... | Small-pox ... | 2 " ... | " F |
| " | 30 .. | Diphtheria ... | 3 " .. | " G |
| Apl. | 8 ... | Scarlet Fever ... | 8 " ... | " D |
| " | 15 ... | " " ... | 1 " ... | " H |
| " | 28 ... | Diphtheria ... | 3 " ... | " A |
| May | 2 ... | Scarlet Fever ... | 5 " ... | " I |
| " | 17 ... | Small-pox ... | 5 " ... | " K |
| Jun. | 26 ... | Scarlet Fever ... | Still engaged ... | " B |
| " | 27 ... | " " ... | " " ... | " H |
| Jly. | 1 ... | " " ... | " " ... | " F |

### ROBBEN ISLAND: A FORTNIGHT AMONGST THE LEPERS.

A VISITOR writes: Circumstances prevented me from labouring very long amongst the lepers on Robben Island, but I cannot speak too highly of the work of the nurses, who devoted their whole time to tending and caring for these poor creatures. Leprosy is a disease which takes many forms, very few of the sufferers being free from ulcerating sores. Four mornings in each week are devoted to dressing these wounds, as many as 200 different patients coming to be attended to. Owing to the great care taken, plenty of the wounds heal and remain well for some time. Loss of eyesight is very common, and it seemed to me to be the saddest misfortune of all. There is a schoolmaster, Melinga by name, paid by Government to instruct the lepers. Every morning the bell rings, and all the pupils, some old and some young, assemble in the day room adjoining the wards. Services are held on week nights and on Sunday mornings. It is a most pathetic sight to see the patients hobbling along on crutches or crawling on all fours, the blind being led in. It is sad to remember that they are incurable, and a nurse needs a cheerful disposition to carry her through her work on Robben Island. Most of the lepers are black, and some do not understand a word of English. Amusements are badly needed, such as games of all sorts, puzzles especially, and plenty of picture books. People at Cape Town send welcome gifts of fruit. It is difficult to maintain harmony amongst Zulus, Basutos, Kaffirs, and Malays, but there has been far less discord since the English chaplain, Mr. Watkins, took up his residence on the island.

### Queen Victoria's Jubilee Institute for Nurses.

HER MAJESTY has been pleased to approve the following additional names being entered on the roll of Queen's nurses for nursing the sick poor in their own homes:—

SUPERINTENDENTS.

Sarah L. Peter (assistant to the inspector), London; Maria B. Vickers, Bangor; Wilhelmina E. Dodds, Darlington.

NURSES.

*England*: Emma E. Jackson, South London; Robina J. Macintyre, Walworth; Amy L. Pratt, Kensington; Clara Warner, Tunbridge Wells; Ellen B. Frederick, Tunbridge Wells; Emma C. Wilks, Haggerston; Emily Basan, Camberwell; Louisa K. Dover, Chelsea; Constance E. Mansel, Ryde; Esther Chadwick, Ryde; Euphemia Moncrieffe, Dulwich; Elizabeth E. Beddy, Gateshead-on-Tyne; Ethel M. Green, Windsor; Emily A. Handy, Banbury; Addy Stewart, Manchester; Ellen Rumsey, Manchester; Mary A. Willott, Manchester; Susannah M. Rose, Bethesda. *Rural District Branch*: Helen Josephine Nathan, Marske-by-the-Sea; Mary S. Rotten, Chesham; Charlotte Podmore, Marple; Jane Helen Marshall, Bradford Perevil; Louisa Metcalf, Honingham; Sydney C. Mourilyan, Highweek; Sarah A Walker, Netheravon; Annie Wilson, Writtle; Elizabeth A. Tite, Spaxton; Alice M. Very, Swimbridge; Ellen Whittingdon, Shenfield; Mary Anne Little, Ingrave; Mary Brogden, Newtown; Matilda Cleverley, Haverland; Helen Hipwell, Quedgeley; Alice R. Sleigh, Burnham Beeches; Sarah E. Crews, Fittleworth; Ada L. Cloutte, Haverfordwest; Alice Mills, Mortimer; Florence Moore, Cleverly Mortimer.

*Wales.*—Ellen Ann Browne, Cardiff; Harriet Cavell, Cardiff.

*Ireland.*—Henrietta M. Warburton, Dublin; Edith M. Golding, Dublin; Harriet Broe, Dublin; Lilian H. Davidson, Ballymena; Norah Diamond, Curragh Camp.

*Scotland.*—Sarah Buchanan, Glasgow; Ann Ellinor Hughes, Glasgow; Janet M'Farlane Paton, Kilmarnock; Margaret M'Nab, Castle Douglas; Ellen Esther Wilson, Peebles; Amelia Marion White, Galashiels; Emily Jesse Hill, Berwick-on-Tweed; Margaret Junner, Anstruther; Katherine Frost, Greenock; Lilian Hall, Lochwinnoch; Agnes Tait, Buckie; Margaret Rutherford, Blairgowrie; Nona Marian Musson, Dumfries; Margaret Brown, Kirriemuir; Isabella M'Dougall, Dundee; Jessie Reid Mitchell, Dundee; Elizabeth Birrell, Dundee; Mary Colvin, Paisley.

### Minor Appointments.

ROYAL HOSPITAL, BELFAST.—Miss B. A. Smallhorn has been made Night Superintendent at the Royal Hospital, Belfast. She was trained at the Taunton and Somerset County Hospital, and was afterwards charge nurse at the Metropolitan Fever Hospital, and sister at Macclesfield Infirmary. We wish Miss Smallhorn every success in her new work.

STANLEY HOSPITAL, LIVERPOOL.—Miss Yarrow has been appointed Sister of the women's wards at the Stanley Hospital. She was trained at the Royal Southern Hospital, Liverpool, and afterwards worked at the Ship Canal Hospital, Ellesmere Port, and at the South-Eastern Hospital, London, and subsequently had charge of the out-patient department at the Royal South Hants Infirmary, Southampton. We wish Miss Yarrow success in her new post. Miss Sherring has been made Sister of the male wards and theatre of the above hospital. She was trained at the Royal Southern Hospital, and afterwards worked in the Southport Infirmary, the South-Eastern Fever Hospital, London, and was for two years Sister of the children's wards, Royal South Hants Infirmary. We wish Miss Sherring every success.

### Nursing in Workhouse Infirmaries.

### A NURSE'S QUALIFICATIONS.

Those who have had experience in the choosing and educating of women for private and district nursing, will agree that a different type is needed for these two spheres of work, and in training very special attention should be given to points which seem of less importance for the hospital nurse. While this is now generally recognised, it is not sufficiently known that for workhouse infirmaries yet another combination of qualifications is needful, and the capabilities of the workhouse nurse have to be exercised under conditions of peculiar difficulty and trial. Just now, when the leaven of Miss Twining's great scheme is working in every direction, and the smaller workhouse infirmaries are seeking skilled nursing for their sick, this is specially the case. A nurse finds herself quite isolated, and the loneliness of her position is in itself a very great trial.

It would be well for those who think of undertaking work for the Workhouse Infirmary Nursing Association earnestly to consider what they need besides the practical and theoretical training they receive in a hospital. Many nurses do not keep a definite end in view during their training. They accept the teaching and practice that come in their way from day to day, but make no steady personal effort for their own individual development, and it is this which is so important for those who are devoting themselves to the pauper sick. Of course there is no quality needed for the workhouse nurse which will not also benefit the hospital, private, and district nurse, but weakness will not show so quickly in other branches as it will in infirmary work.

If a patient is kept in a hospital ward for any unusual time, it is only too common for the nurse's interest to flag. "Just an old chronic," she says, and though she may honestly do her duty for that case, her want of interest is evident; she is less cheerful and bright, and the patient is painfully conscious of this. What an opportunity this is for the nurse who is training for workhouse work to force herself to take as keen an interest in that poor chronic's wounds as in any show surgical case in the ward. In an infirmary ward she will probably have ten such patients under her care for every one she has in hospital.

In the matter of ward work, in what a different spirit the cleaning and dusting would be done if the nurse could realise the value such practice would be to her in the future, when directing and controlling the pauper ward-women. To hear a nurse speak scornfully or complainingly of "menial" work is always displeasing. Is not every nurse the servant of the sick? No service, however rough, should be despised if it is, in its highest sense, for the sick. "Waste of skilled labour," people are fond of saying; but when once an educated and cultivated woman brings her intelligence to bear on the question of the best methods by which to apply cleansing materials to surfaces on which there is an accumulation of "matter out of place," it is beautiful to see the devices of the intellectual to overcome the want of physical force. The latter, certainly, is often lacking in the lady in comparison with the woman whose muscles have received the development her brain has missed. Nothing raises more quickly the low standard and flagging energies of the workhouse wardwoman than to see the nurse, when necessity arises, turn up her sleeves and set her the example of quick, thorough work, thereby overcoming the difficulty which, if left to her untrained hands, would have resulted in discomfort to the patients and accumulation of work.

No woman can always do uniformly good work, but as the best tempered steel has the most spring in it, so the nurse who is not only the best hospital-trained, but also the best self-trained, will have an amount of rebound which will keep her quick-witted and cheerful in spite of discouraging and depressing surroundings. Nowhere is the necessity for the nurse being "all there" greater than in a workhouse ward. To maintain discipline; prevent the squabbling so apt to break out among dozens of old women; to check the grumbling, which is the solace of the men's day-rooms; to be up to the ways by which the wardswomen and men shirk work and break rules; to forestall the difficulties that will occur with officials and guardians; all these require great alertness of mind on the part of the nurse. Her view of the situation must be broad and kindly, and her heart must be full of unselfish cheerfulness. Many may begin well, but time is the trial. Unless unselfishness is rooted in the highest principles, the monotony of workhouse routine, the presence of red-tapeism, and the small worries will prove fatal, and the nurse will either change to more hopeful work, or else sink into a mere grumbling machine. And what a sad failure that is! What possibilities for herself and others would be lost? Faced in the right spirit, the work need never be dull. If small things are glorified by the highest motives, they will never be monotonous. While the movement is young and the difficulties of first starting skilled workers to succeed the Gamps of the olden time is great, the very best workers of the nursing world are wanted for quite small changes. The pay is poor, but surely there are women who are noble enough to lay aside the ambition of place and money, and give their brains and skill to the poorest of the poor. The better educated a woman is, the wider her own mental grasp, the better she will economise labour, and by wise organisation get the best results, two points most important in every detail of institution work, and the more her own development is many-sided, the better field she has for interesting and inspiring with some brightness the lives around her. This is surely an end worth working for. In order to bring the mere elements of humanity and a minimum of necessary hygenic principle to bear on the child, aged, and sick paupers of the country, the difficulties to be overcome are still, though much has been done, enormous. Good and wise women are needed for the work, but goodness and wisdom will not avail without courage also. A strong, quiet confidence in the ultimate triumph of the object they have at heart will alone carry them through the opposition and delays with which every attempt at reform will be met. It would be well if every nurse before entering a workhouse infirmary would face the position and have no illusions on the subject, and then, having put her hand to the plough, never look back but forward to the blessed harvest which she, indeed, may never live to see, but to which she will surely come.

### Notes and Queries.

### SPECIAL NOTICE.

The contents of the Editor's Letter-box have now reached such unwieldy proportions that it has become necessary to establish a hard and fast rule regarding Answers to Correspondents. In future, all questions requiring replies will continue to be answered in this column without any fee. If an answer is required by letter, a fee of half-a-crown must be enclosed with the note containing the enquiry. We are always pleased to help our numerous correspondents to the fullest extent, and we can trust them to sympathise in the overwhelming amount of writing which makes the new rules a necessity.

### Queries.

(153) *Dispensing.*—How can a woman learn dispensing?—*L.H.*

(154) *Flowers.*—Can anyone give me address of a society which distributes flowers to the poor?—*Flora.*

(155) *India.*—Can you give me the name of the hon. sec. to the Countess of Dufferin's Fund?—*Novice.*

(156) *Pulse.*—I shall be glad to know of a book on the pulse.—*Anxious Enquirer.*

### Answers.

(153) *Dispensing* (*L.H.*)—There is a chapter on this subject which explains everything you ask about, in Burdett's Hospital Annual published by Scientific Press, 428, Strand.

(154) *Flowers* (*Flora*).—Hon. sec. Flower Distribution, Kyrle Society, 49, Manchester Street, London.

(155) *India* (*Novice*).—The Countess of Malmesbury is the hon. sec., and Messrs. Coutts, 59, Strand, are bankers for Lady Dufferin's fund.

(156) *Pulse* (*Anxious Enquirer*).—"How to Feel the Pulse," by W. Ewart, can be ordered from Messrs. Lewis, Gower Street, or any other medical bookseller.

### The Book World for Women and Nurses.

[We invite Correspondence, Criticism, Enquiries, and Notes on Books likely to interest Women and Nurses. Address, Editor, The Hospital (Nurses' Book World), 428, Strand, W.C.]

Crime and Punishment. By Fedor Dostoieffsky. (Walter Scott, London.)

This is a new edition of a book that has been long before the public, but although it has attracted a good deal of attention in some quarters, it cannot be said to have become generally popular. Those readers who selected it possibly from the *pot pourri* of new publications on a bookstall desiring to while a few hours away agreeably only, may be excused if they failed to appreciate the real value of the book. The title leads them to expect the purely sensational, whilst they find themselves introduced to morbid processes of introspection and analysis which clog the wheels of the story. To the ordinary English nature this intensity and morbid wretchedness with which the book abounds appears wholly unnatural and exaggerated. Those who are more familiar with the Sclavonic temperament know that this is not the case. Dostoieffsky belonged to a race stronger for evil than our own, stronger in their desires, and stronger in their power of suffering. Surrounded by beings living under far less happy social conditions than ours, he himself was especially familiar with the sad and tragic in life. His childhood was acquainted with the aspect of suffering, bred as he was at the Hospital for the Poor at Moscow, where his father was attached as doctor. His temperament, a naturally sensitive one, was sorely tried by the struggle for existence, which he entered after the death of his father. He then became acquainted with the lives and feeling of the poor classes, a fact that is evident in his writings. He is described as having the soul of a woman in the envelope of a Russian peasant. The feminine element in his character none reading his writings intelligently can fail to recognise. There are subtle points of feeling, and an attention to the sentiment of small things seldom found in the works of masculine writers. "Crime and Punishment," gives a very real picture of one phase of Russian life, and morbid and wretched as the general impression given by the writer is, nevertheless it is his actual experience which is chiefly answerable. Dostoieffsky takes for his hero a young student studying at Moscow, whom he depicts as possessing a highly strung temperament, probably not unlike his own. He has very limited means; all his widowed mother can afford to give him. In seeking for the true conduct of life, he falls into a quagmire of confusion. He commits the crime of murder, but whilst he reasons that he has done a good deed in ridding the world of a being who existed but to the harm of others, he is haunted with all the horrors and terrors of one not hardened to crime. There is nothing to betray the unfortunate Raskolnikoff, and no suspicion falls on him; but mind and body alike give way to the strain, for reason fails to reconcile him to the deed, and he runs every risk of betraying himself. Stories of the lives of those with whom he comes in contact are also told. Most are tinged with misfortune in some form or other. The characters of his sister Dounia, and that of an unfortunate girl, Sonia, sacrificed to the wants of her family, are the most pleasing in the book. Each are strong in their way, and both love him.

### THE BOOK WORLD FOR WOMEN AND NURSES—*continued.*

Both inspire him with courage to expiate his fault and confess, for the crime is revealed to both. Raskolnikoff is exiled to Siberia. Sonia follows him, and in spite of the wretchedness of his lot, through her love restores the balance to his unsettled mind. In the words of the book, "A new history commences, a story of the gradual renewing of a man, of his slow progressive generation, and change from one world to another—an introduction to the hitherto unknown realities of life." This climax forms a great relief to feelings harrowed by the sadder commencement of the romance. We have given the briefest outline of a book that is full of the strongest human interest, and will well repay all but the wandering attention of the casual reader. For such it is not suited.

---

LYRICS. By J. A. GOODCHILD. (Horace Cox. 5s.)

THIS volume of short poems is stated by the author to be a selection of miscellaneous pieces from works already published. They are of very unequal merit, but the reader who turns the pages attentively, and is not discouraged by opening first upon verse like "Pearls," for instance, or a note of narrow insight such as is displayed in "Sister Seraphina," may wander pleasantly at will and find many suggestive thoughts and much beauty of expression. The key-note of them all is faith. If the writer is ever intolerant it is towards intolerance, or towards a certain vulgarity of worldly-mindedness. Deducting these obnoxious traits, which he cannot away with, his sympathies are broad, and in its perception of the soul of good in things evil, his verse is a wholesome and invigorating example among the crowd of minor poets who merely skim the surface. The "Valley of Shadow," a beautiful piece of descriptive writing, realises the very spirit of desolation, "Judge Not," the essence of degradation, yet both are relieved by the perception of good surviving in the degradation, and accessible even from the valley of desolation itself. And this faith illuminates a quiet pathos, bearing witness to large experience of sorrow and suffering in others. The "Swallows of the Riviera" is very touching in its suggestion of long observation of

The sick, the weary, pale, and ghostly bands
Who gather sunshine by the southern sea.

There is record of kindly observation in the charming little sketch in few lines of hidden lives passed in

Fair, sweet, and quiet rest; and fruitfulness.

We quote the sonnet "Crippled" as an example of this strength in pathos:

Her days were spent in prison, for disease
Gat hold upon her youth, and vexed her sore
With pain's disablement, and shut the door
Of her outgoings; but within dwelt peace.
Patient she watched the sluggish years increase
The weary chains of feebleness she wore,
And, unreluctant, bowed her head before
The appointed angel of her soul's release.
She brings her gift of patience; all *she* had,
Who lacked her childhood's sports and youth's delight,
Who drank her cup in silence; but, when bade,
Entered into the Temple and was glad.
And He who takes her offering will not slight
Her toll, but store it with the widow's mite.

